# Circular RNA circSLC8A1 acts as a sponge of miR-130b/miR-494 in suppressing bladder cancer progression via regulating PTEN

**DOI:** 10.1186/s12943-019-1040-0

**Published:** 2019-06-22

**Authors:** Qun Lu, Tianyao Liu, Huijin Feng, Rong Yang, Xiaozhi Zhao, Wei Chen, Bo Jiang, Haixiang Qin, Xu Guo, Minghui Liu, Limin Li, Hongqian Guo

**Affiliations:** 1 Department of Urology, Drum Tower Hospital, Medical School of Nanjing University, Institute of Urology, Nanjing University, 321 Zhongshan Road, Nanjing, 210008 Jiangsu China; 2 NJU Advanced Institute for Life Sciences, Jiangsu Engineering Research Center for MicroRNA Biology and Biotechnology, Nanjing University, 163 Xianlin Avenue, Nanjing, 210023 Jiangsu China

## Abstract

**Background:**

Circular RNAs (circRNAs) are a novel class of endogenous noncoding RNAs formed by a covalently closed loop, and increasing evidence has revealed that circRNAs play crucial functions in regulating gene expression. CircSLC8A1 is a circRNA generated from the *SLC8A1* gene. Currently, the role and underlying molecular mechanisms of circSLC8A1 in bladder cancer remain unknown.

**Methods:**

The differentially expressed circRNAs were identified from RNA-sequencing data, and circSLC8A1 was determined as a new candidate circRNA. qRT-PCR was used to detect the expression of circRNAs, miRNAs and mRNAs in human tissues and cells. RNA pull-down assay and luciferase reporter assay were used to investigate the interactions between the specific circRNA, miRNA and mRNA. The effects of circSLC8A1 on bladder cancer cells were explored by transfecting with plasmids in vitro and in vivo. The expression of PTEN was detected by Western blot. The biological roles were measured by wound healing assay, transwell assay, and CCK-8 assay.

**Results:**

In the present study, we found that circSLC8A1 was down-regulated in bladder cancer tissues and cell lines, and circSLC8A1 expression was associated with the pathological stage and histological grade of bladder cancer. Over-expression of circSLC8A1 inhibited cell migration, invasion and proliferation both in vitro and in vivo. Mechanistically, circSLC8A1 could directly interact with miR-130b/miR-494, and subsequently act as a miRNA sponge to regulate the expression of the miR-130b/miR-494 target gene *PTEN* and downstream signaling pathway, which suppressed the progression of bladder cancer.

**Conclusions:**

CircSLC8A1 acts as a tumor suppressor by a novel circSLC8A1/miR-130b, miR-494/PTEN axis, which may provide a potential biomarker and therapeutic target for the management of bladder cancer.

**Electronic supplementary material:**

The online version of this article (10.1186/s12943-019-1040-0) contains supplementary material, which is available to authorized users.

## Background

Bladder cancer is the most common malignancy of the urinary system and is one of the most prevalent malignancies worldwide [1]. In China, the mortality and morbidity of bladder cancer ranked first among all the tumors of urinary system [2]. Bladder cancer can be classified into two types according to the depth of tumor invasion: non-muscle invasive tumor (70 ~ 80%) and muscle-invasive tumor (20~30%) [3].For the patients with muscle-invasive bladder cancer, the occurrence of metastasis is more frequent, and the prognosis is poorer [4]. Even in those muscle-invasive bladder cancer patients who receive optimal treatment with surgery and chemotherapy, the 5-year overall survival rate is only 60% due to distant metastasis [5]. Therefore, it is of great clinical significance to clarify the molecular mechanisms that drive the progression of bladder cancer, which will help to develop more effective anticancer therapies.

Circular RNA (circRNA) is a novel class of endogenous noncoding RNA molecules generally characterized by a covalently closed loop structure without a 5′ cap and a 3′poly A tail [6]. Unlike linear RNAs, circRNAs usually originate from back splicing events of exons or introns. Reversed complementary sequences including inverted repeated Alu pairs and exon skipping are essential to circRNA formation [7, 8]. Salient features of circRNAs include significant stability, high abundance, evolutionary conservation, and tissue-specific expression [9, 10]. Although circRNAs were reported many years ago [11], these molecules were first considered as products of splicing errors [12]. Genome-wide analyses of RNA sequencing data have identified large amounts of circRNAs and proven that they are endogenous, abundant and conserved in mammalian cells, suggesting specific roles of circRNAs in cellular physiology [6, 9]. Many circRNAs have been demonstrated to have critical roles in the carcinogenesis and development of cancers [13, 14].

MicroRNAs (miRNAs) are endogenous small noncoding RNA molecules (19–22 nucleotides in length) that negatively modulate the expression of protein-coding genes through binding to the specific sequence of genes [15]. Increasing evidence suggests that miRNAs are aberrantly expressed in bladder cancer and they play significant roles in the tumorigenesis, development, and metastasis of cancer [16]. Multiple properties of circRNAs have been identified in recent years, among which the role of “miRNA sponges” was most frequently discussed since some circRNAs possess miRNA binding sites [17]. CircRNAs sequester miRNAs to terminate the regulation of their target genes [18]. Currently, there are few reports describing the role of circRNAs in bladder cancer [19–21]. The biological functions of circRNAs in bladder cancer remain largely unknown and require further investigation.

Here, we identified a circRNA derived from the *SLC8A1* gene, termed circSLC8A1. The expression of circSLC8A1 was significantly down-regulated in bladder cancer tissues and cell lines, and it was positively correlated with the clinical stage and grade of bladder cancer. Therefore, we presented a hypothesis that circSLC8A1 might be involved in the progression of bladder cancer, via sponging miR-130b and miR-494 to influence the expression of PTEN. Collectively, circSLC8A1 may serve as a promising target for bladder cancer treatment.

## Methods

### Cell lines and human tissues

The human bladder cancer cell lines (5637, T24, J82, EJ, UMUC, and RT4) and the human urothelial epithelial cell line (SV-HUC-1) were purchased from the Shanghai Institute of Cell Biology, Chinese Academy of Sciences (Shanghai, China). The cells were cultured in RPMI 1640 medium supplemented with 10% fetal bovine serum (FBS, Gibco, Carlsbad, CA, USA), 100 units/ml penicillin and 100 μg/ml streptomycin in a humidified atmosphere containing 5% CO2. The bladder cancer specimens and paired normal adjacent tissues were obtained from patients undergoing a surgical procedure at the Affiliated Drum Tower Hospital of Nanjing University Medical School (Nanjing, China). All the patients provided written consent, and the Ethics Committee from Nanjing University approved all aspects of this study. Tissue fragments were immediately frozen in liquid nitrogen at the time of surgery and stored at − 80 °C.

### RNA isolation and quantitative RT-PCR (qRT-PCR)

Total RNA was extracted from cultured cells and human tissues using Trizol reagent (Sigma, St. Louis, MO, USA) according to the manufacturer’s instructions. Reverse transcription of mRNA and miRNA was conducted using random primers and stem-loop primers in TaKaRa system (Dalian, China), respectively. Real-time PCR was performed using a TaqMan Universal Master Mix II kit on an Applied Biosystems 7500 Sequence Detection System (Applied Biosystem). The levels of target genes were calculated based on the cycle threshold (Ct) values compared to a reference gene using the formula 2^-∆∆Ct^. GAPDH mRNA and U6 snRNA were used as references for mRNA and miRNA, respectively. The details of primers were listed in Additional file 4: Table S2.

### Protein extraction and Western blotting

The cells and tissues were lysed in ice-cold RIPA lysis buffer (Beyotime, Shanghai, China) supplemented with 1% PMSF, incubated on ice for 30 min and then centrifuged for 10 min (12,000×g, 4 °C). The supernatant was collected, and the protein concentration was calculated using a Pierce BCA protein assay kit (Thermo Scientific, Rockford, IL, USA). The PTEN protein levels were analyzed by Western blotting with an anti-human PTEN antibody (9559, Cell Signaling Technology, MA, USA). The protein levels were normalized by probing the same blots with a β-actin antibody (05–0079, AbMax, Beijing, China).

### Plasmid construction and siRNA interference assay

To construct circSLC8A1 over-expression plasmids, human circSLC8A1 cDNA was synthesized and cloned into a pLVX-cir vector by Genomeditech (Shanghai, China), which contained a front circular frame and a back circular frame. An empty plasmid served as the negative control. Two siRNA sequences were synthesized by GenePharma (Shanghai, China). A scrambled siRNA was synthesized as a negative control. Transfection was carried out using Lipofectamine 3000 (Invitrogen) according to the manufacturer’s instructions. Total RNA and protein were collected 48 h after transfection.

### Wound healing assay

Cells were seeded in 6-well plates with 5 × 10^5^ cells per well. Then, a wound was made by using a 200 μl pipette tip on the cell monolayer and photographs were taken at the appropriate time to estimate the area occupied by migratory cells.

### Transwell assay

Transwell (Costar, New York, NY, USA) assay was used to evaluate the invasion and migration capacities of bladder cancer cells in vitro. Cells at a concentration of 1 × 10^5^ cells in 500 μl of serum-free medium were inoculated in the upper chamber, coated with (invasion assay) or without (migration assay) growth factor reduced Matrigel®, and medium containing 10% FBS was added into the lower chamber as a chemoattractant. After incubation for the appropriate time, cells on the upper surface of the membrane were removed by wiping with a Q-tip, and the invaded or migrated cells were fixed with formaldehyde and stained using 0.5% crystal violet (Sigma). The numbers of invaded and migrated cells were counted in five randomly selected fields under a microscope.

### Cell proliferation assay

The proliferation of bladder cancer cells was determined using the Cell Counting Kit-8 (CCK-8, Dojindo, Japan) according to the manufacturer’s instructions. 5637 and T24 cells were plated at a density of 5 × 10^3^ cells per well in 96-well plates. At the indicated time points, the cells were treated with 10 μl of CCK-8 solution (Dojindo, Japan) and incubated in the dark for another 2 h. The absorbance was measured at a wavelength of 450 nm.

### Pull-down assay with biotinylated circSLC8A1 probe

The biotinylated probe was specifically designed to bind to the junction area of circSLC8A1, while the oligo probe was taken as a control. Approximately 1 × 10^7^ cells were harvested and lysed. The circSLC8A1 probe (Tsingke, Wuhan, China) was incubated with streptavidin magnetic beads (Life Technologies, USA) at room temperature for 2 h to generate probe-coated beads. The cell lysates were incubated with probe-coated beads at 4 °C overnight. The beads were washed and the bound miRNAs in the pull-down materials were extracted using Trizol reagent and analyzed by qRT-PCR assay.

### Pull-down assay with biotinylated miRNA

Bladder cancer cells were transfected with 50 nM of biotinylated miRNA mimics or nonsense control (Tsingke, Wuhan, China) at 50% confluence using Lipofectamine 3000 (Invitrogen). The cells were harvested and lysed in lysis buffer 24 h after transfection. The cell lysates were incubated with washed streptavidin magnetic beads (Life Technologies) for 3 h. The beads were washed and Trizol reagent was used to extract RNA interacting with miRNA. The abundance of circSLC8A1 was evaluated by qRT-PCR analysis.

### Fluorescence in situ hybridization (FISH)

Cy3-labeled probes were specific to circSLC8A1 and fam-labeled probes were specific to miR-130b/miR-494. The probes were designed and synthesized by Genepharma (Shanghai, China), and the signals of the probes were detected by a Fluorescent In Situ Hybridization Kit (Genepharma, Shanghai, China) according to the manufacturer’s instructions. The images were acquired on Lei TCS SP8 Laser Scanning Confocal Microscope (Leica Microsystems, Mannheim, Germany).

### Luciferase reporter assay

A sequence containing the presumed binding sites of miR-130b and miR-494 was designed from the human *PTEN* 3′-UTR. The sequence was inserted into the p-MIR-reporter plasmid. The insertion was confirmed to be correct by sequencing. To test the binding specificity, the sequences that interacted with the miR-130b/miR-494 seed sequence were mutated, and the mutant *PTEN* 3′-UTR was inserted into an equivalent luciferase reporter. For the luciferase reporter assays, HEK293 cells were cultured in 24-well plates, and each well was transfected with 0.4 μg of firefly luciferase reporter plasmid; 0.4 μg of a β-galactosidase (β-gal) expression plasmid (Ambion); and equal amounts (20 pmol) of mim-miR-130b/mim-miR-494, or mim-miR-control using Lipofectamine 3000 (Invitrogen). The β-gal plasmid was used as a transfection control. Twenty-four hours after transfection, the cells were assayed using a luciferase assay kit (Promega, Madison, WI, USA).

### Immunohistochemistry (IHC)

The primary antibody used to detect PTEN was purchased from Cell Signaling Technology (Beverly, MA, USA), and the primary antibodies used to detect Akt, p-Akt, and MMP-9 were purchased from Proteintech Group (Chicago, IL, USA). The immunostaining images were captured using a microscope (Leica, Germany). The immunoreactivity in each tissue section was assessed by at least two pathologists. The degree of positivity was determined according to the percentage of positive tumor cells.

### Establishment of tumor xenografts in mice

Four-week-old female nude mice were purchased from the Model Animal Research Center at Nanjing University (Nanjing, China) and maintained under specific pathogen-free conditions at Nanjing University. The mice were randomly divided into 2 groups and subcutaneously injected with T24 cells (5 × 10^6^ cells per mouse, 6 mice per group) that were stably transfected with circSLC8A1 plasmids or control vector. Tumor growth was monitored every week by measuring the width (W) and length (L) with calipers, and the volume (V) of the tumor was calculated using the formula V = (W^2^ × L)/2. The mice were sacrificed after four weeks. The tumors were excised, and the tumor weight was measured. Portions of the tumor samples were used for total RNA extraction, and the remainder were fixed in 4% paraformaldehyde for 24 h and then processed for hematoxylin and eosin (H&E) staining as well as IHC staining.

### Statistical analysis

Data are presented as the mean ± standard deviation (SD) from three independent experiments unless otherwise noted. A paired *t*-test was used to analyze the differences in circSLC8A1 and miR-130b/miR-494 levels between cancer tissues and corresponding normal tissues. Other differences between the two groups were analyzed using the Student’s *t*-test or Chi-square test. The Pearson’s correlation coefficient analysis was used to analyze the correlations. A *p*-value < 0.05 was considered statistically significant.

## Results

### circSLC8A1 (hsa_circ_0000994) is significantly down-regulated in bladder cancer

**Fig. 1:**
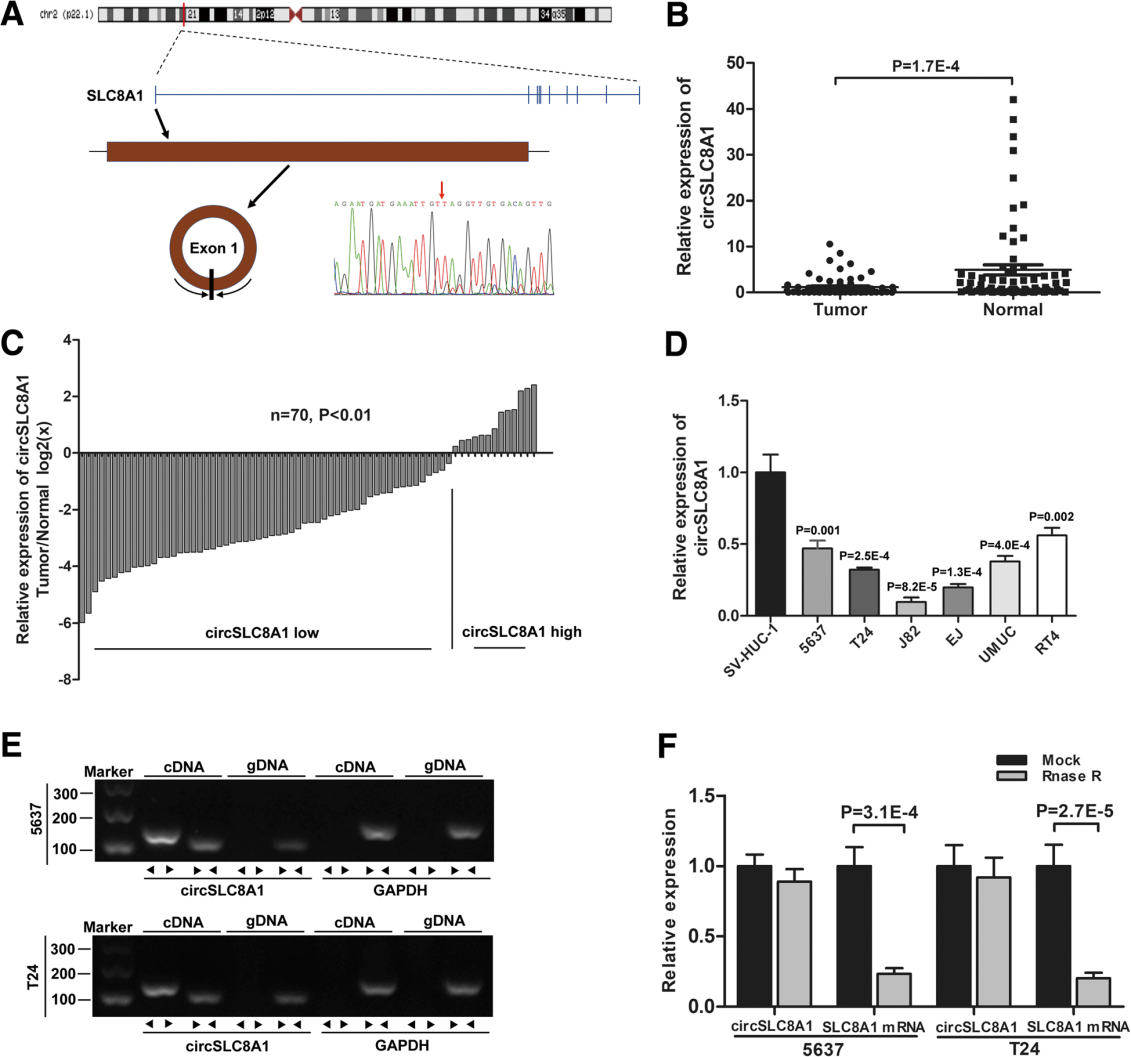
circSLC8A1 is significantly down-regulated in bladder cancer tissues and cell lines. **a** Schematic illustration showing the circularization of *SLC8A1* exon 1 forming circSLC8A1. The existence of circSLC8A1 was proved by RT–PCR and its back splicing junction was verified by Sanger sequencing. Red arrow indicates the special splicing junction of circSLC8A1. **b** and **c** qRT-PCR assay with divergent primers confirmed the low expression of circSLC8A1 in 70 pairs of human bladder cancer tissues compared with their adjacent normal tissues. **d** The expression of circSLC8A1 in SV-HUC-1 and bladder cancer cell lines were measured by qRT-PCR. **e** The existence of circSLC8A1 was validated in 5637 and T24 cell lines by RT–PCR. Divergent primers amplified circSLC8A1 in cDNA but not genomic DNA (gDNA). GAPDH was used as negative control. **f** The expressions of circSLC8A1 and *SLC8A1* mRNA in 5637 and T24 cells treated with or without RNase R were detected by qRT-PCR. The relative levels of circSLC8A1 and *SLC8A1* mRNA were normalized to the value measured in the mock treatment. Data are mean ± SD, *n* = 3

To identify the role of circRNAs in the development of bladder cancer, differentially expressed circRNAs were studied from the RNA sequencing data of a previous study [19]. Among the differentially expressed circRNAs, down-regulated circRNAs are more common than up-regulated circRNAs. We screened the down-regulated circRNAs with an average normal tissue read count of more than 100 and then sorted them by fold change. The five most down-regulated circRNAs were validated using qRT-PCR in 20 pairs of bladder cancer tissues and matched adjacent normal tissues, and the details of five circRNAs were listed in Additional file 3: Table S1. The results showed that two of the five circRNAs were significantly down-regulated in bladder cancer tissues, and circSLC8A1 (hsa_circ_0000994) was the most down-regulated circRNA according to the qRT-PCR results (Additional file 1: Figure S1). circSLC8A1 arose from the *SLC8A1* gene and consisted of the head-to-tail splicing of exon 1 (1832 bp) (Fig. 1a). According to the RNA-sequencing result, circSLC8A1 was down-regulated in bladder cancer tissues (Log2FC = 5.28). Moreover, we also found that circSLC8A1 was down-regulated in bladder cancer from another RNA-sequencing data set (Log2FC = 3.31) [22], and circSLC8A1 is abundant in early-stage bladder cancer tissues [23]. Subsequently, we confirmed the head-to-tail splicing in the RT-PCR product of circSLC8A1 with the expected size by Sanger sequencing (Fig. 1a). To investigate the expression of circSLC8A1 in bladder cancer, we detected the expression level of circSLC8A1 in 70 pairs of bladder cancer tissue and normal bladder tissue, and the results confirmed that circSLC8A1 had significant low expression in bladder cancer tissues (Fig. 1b). Notably, paired analysis of bladder cancer tumor samples showed that the expression of circSLC8A1 was decreased in more than 80% of all the patient samples (Fig. 1c). Also, the correlation analysis showed that circSLC8A1 expression was associated with clinicopathological features including the pathological stage and histological grade (Table 1). Additionally, circSLC8A1 showed a lower expression in 6 bladder cancer cell lines (5637, T24, J82, EJ, UMUC, and RT4) compared to SV-HUC-1, which is a normal urothelial cell line (Fig. 1d). Next, we designed convergent primers to amplify *SLC8A1* mRNA and divergent primers to amplify circSLC8A1. Using cDNA and gDNA (genomic DNA) from 5637 and T24 cell lines as templates, circSLC8A1 was only amplified by divergent primers in cDNA, and no amplification product was observed in gDNA (Fig. 1e). By using qRT-PCR, we further confirmed that circSLC8A1 was resistant to RNase R, while *SLC8A1* mRNA was significantly reduced after RNase R treatment (Fig. 1f).

**Table 1:** Correlations between the expression of circSLC8A1 and clinicopathological features in 70 bladder cancer patients

	Cases	circSLC8A1 expression		*p* Value
		Low	High	
Age				0.687
< 55	21	16	5	
≥55	49	41	8	
Gender				1.000
Male	52	42	10	
Female	18	15	3	
T stage				< 0.001
pTa-T1	19	8	11	
pT2-T4	51	49	2	
Grade				0.006
Low	23	14	9	
High	47	43	4	
Lymph node metastasis				0.057
Absent	43	32	11	
Present	27	25	2	
Total	70	57	13	

### Over-expression of circSLC8A1 inhibits the migration and invasion of bladder cancer cell

**Fig. 2:**
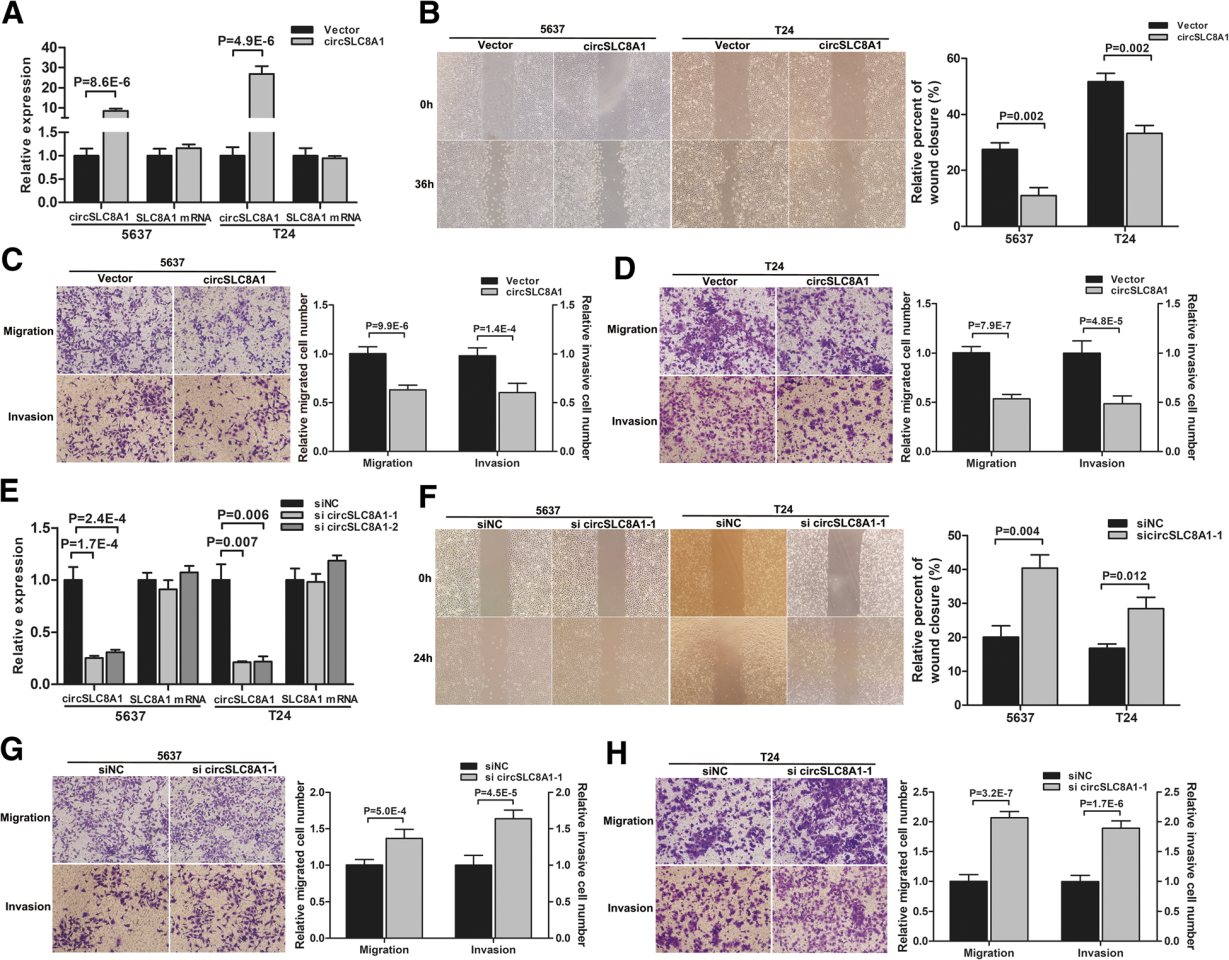
Over-expression of circSLC8A1 inhibits the migration and invasion of bladder cancer cell. **a** The expression levels of circSLC8A1 and *SLC8A1* mRNA in 5637 and T24 cells after transfection with circSLC8A1 or control vector plasmids were detected by qRT-PCR. **b** The effect of circSLC8A1 on cell migration capability was evaluated by wound healing assay in 5637 and T24 cells, respectively. **c** and **d** Cell migration and invasion abilities of 5637 and T24 cells transfected with circSLC8A1 or control vector were evaluated by transwell migration and matrigel invasion assays. **e** Two siRNAs specifically targeting circSLC8A1 were transfected into 5637 and T24 cells. The interfering efficacy of each siRNA on circSLC8A1 and *SLC8A1* mRNA was tested by qRT-PCR. **f** The effect of si circSLC8A1–1 on cell migration capability was evaluated by wound healing assay in 5637 and T24 cells, respectively. **g** and **h** Cell migration and invasion abilities of 5637 and T24 cells transfected with si circSLC8A1–1 or siNC were evaluated by transwell migration and invasion assays. Data are mean ± SD, *n* = 3

Functional assays were conducted to further validate the role of circSLC8A1 in bladder cancer progression. Given that circSLC8A1 is down-regulated in bladder cancer tissues and cell lines in our study, we transfected the circSLC8A1 expression construct into 5637 and T24 cells, and the expression of circSLC8A1 was significantly increased (Fig. 2a). Meanwhile, the expression of *SLC8A1* mRNA had no significant change (Fig. 2a). Wound healing assay revealed that over-expression of circSLC8A1 significantly inhibited cell migration in 5637 and T24 cells (Fig. 2b). Consistently, transwell migration and matrigel invasion assays indicated that the migration and invasion abilities of bladder cancer cell lines were also suppressed by over-expression of circSLC8A1. (Fig. 2c and d). Next, we transfected siRNAs targeting the junction sites of circSLC8A1 into 5637 and T24 cells. These siRNAs significantly decreased the expression of circSLC8A1, but they had no effect on *SLC8A1* mRNA (Fig. 2e). The results of wound healing assay and transwell assay showed that circSLC8A1 knockdown significantly increased the migration and invasion capabilities of 5637 and T24 cells (Fig. 2f–h and Additional file 2: Figure S2). These results suggested that circSLC8A1 played a role as tumor suppressor through inhibiting migration and invasion in bladder cancer cells.

### circSLC8A1 acts as a sponge for miR-130b and miR-494 in bladder cancer cell

**Fig. 3:**
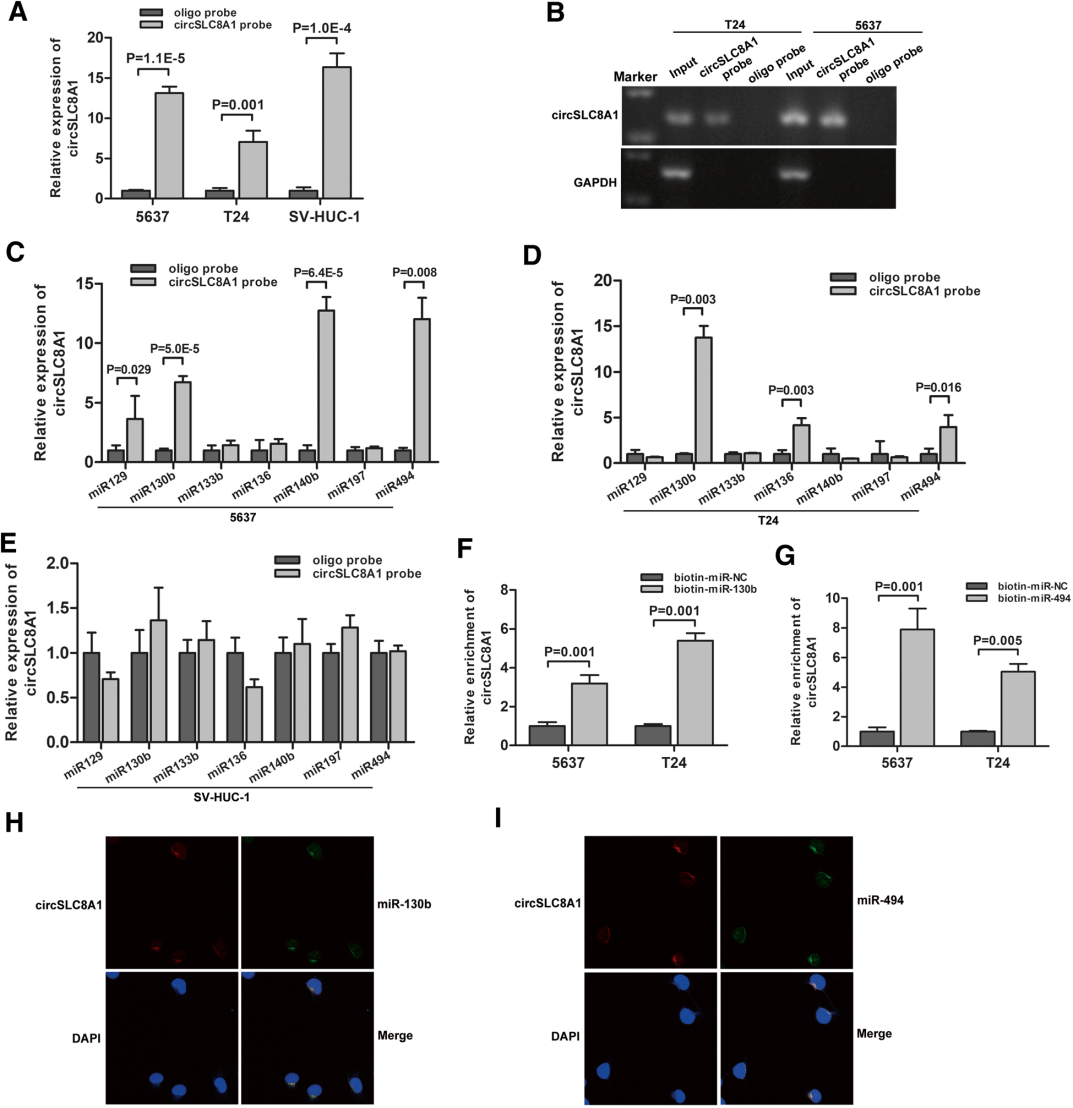
circSLC8A1 acts as a sponge for miR-130b and miR-494 in bladder cancer cells. **a** and **b** circSLC8A1 in the 5637, T24 and SV-HUC-1 lysates was pulled down and enriched with circSLC8A1 specific probe and then detected by qRT-PCR. Relative level of circSLC8A1 was normalized to the input. GAPDH was used as a negative control. **c, d** and **e** The relative levels of 7 miRNA candidates in the 5637, T24 and SV-HUC-1 lysates were detected by qRT-PCR. Multiple miRNAs can be pulled down by circSLC8A1, and both miR-130b and miR-494 were pulled down by circSLC8A1 probe in 5637 and T24 lines. **f** and **g** The biotinylated miR-130b or miR-494 was transfected into 5637 and T24 cells. After streptavidin capture, circSLC8A1 levels were quantified by qRT-PCR. **h** and **i** RNA FISH for circSLC8A1 and miR-130b/miR-494 was detected in T24. Nuclei was stained blue (DAPI), circSLC8A1 was stained red, and miR-130b/miR-494 were stained green. Data are mean ± SD, n = 3

It had been reported that circRNAs function as miRNA sponges to regulate miRNA expression [22]. To address whether circSLC8A1 could act as a sponge for miRNAs in bladder cancer cells, we predicted the possible binding miRNAs of circSLC8A1 by using three publicly available prediction tools miRanda (http://www.microrna.org/microrna/home.do), Circinteractome (https://circinteractome.nia.nih.gov/), or RNAhybrid (https://bibiserv.cebitec.uni-bielefeld.de/rnahybrid/). We selected 7 candidate miRNAs, which were predicted by at least two of the above three prediction tools and previously reported as oncogenic miRNAs. Subsequently, we applied the biotin-labeled probe pull down assay to investigate whether circSLC8A1 could directly bind these candidate miRNAs. The biotin-labeled probe was verified to pull down circSLC8A1 in 5637, T24 and SV-HUC-1 cell lines (Fig. 3a and b). The levels of the 7 candidate miRNAs were detected, and the results revealed that miR-130b and miR-494 were the only two miRNAs that were abundantly pulled down by circSLC8A1 in both 5637 and T24 cells (Fig. 3c, d and e). To further confirm the sponge effect of circSLC8A1, we applied biotin-labeled miR-130b and miR-494 mimics to verify the direct binding of the miRNAs and circSLC8A1. The results showed that biotin-labeled miR-130b/miR-494 captured more circSLC8A1 than the biotin-labeled negative control (Fig. 3f and g). Moreover, RNA FISH assay revealed that circSLC8A1 and miR-130b/miR-494 were co-localized in the cytoplasm (Fig. 3h and i). The above results indicate that circSLC8A1 can directly bind to miR-130b and miR-494.

### miR-130b and miR-494 promote bladder cancer progression through targeting *PTEN*

**Fig. 4:**
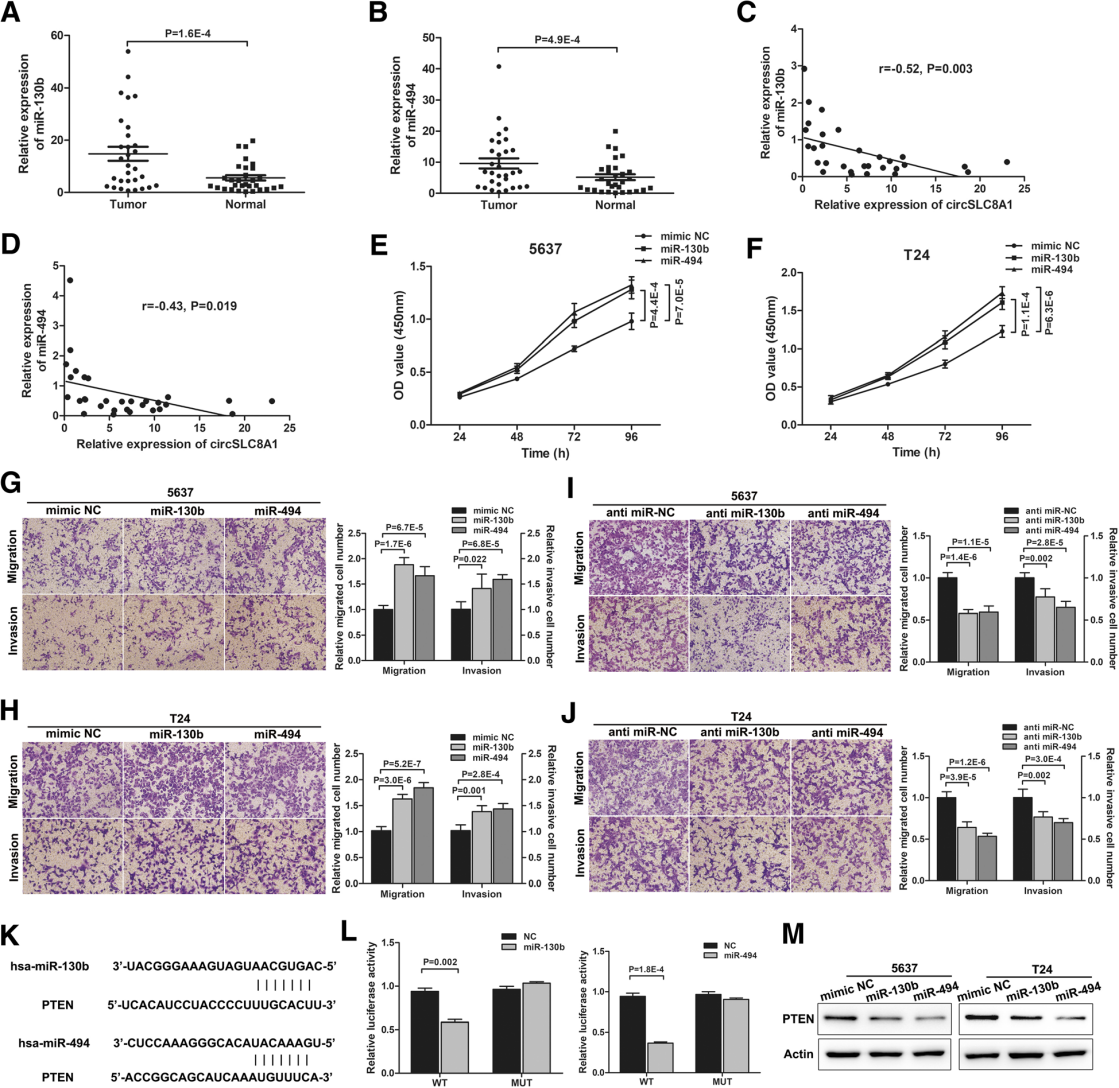
miR-130b and miR-494 promote bladder cancer progression through targeting *PTEN*. **a** and **b** qRT-PCR revealed that miR-130b and miR-494 were up-regulated in bladder cancer tissues (*n* = 30) compared with normal adjacent bladder tissues. **c** and **d** A negative correlation between the expression of circSLC8A1 and miR-130b/miR-494 was showed using Pearson correlation analysis. **e** and **f** CCK-8 assay showed that miR-130b and miR-494 promoted the proliferation of 5637 and T24 cells. **g** and **h** Transwell migration and matrigel invasion assays indicating the increased migration and invasion capabilities in 5637 and T24 cells transfected with miR-130b or miR-494 mimics. **i** and **j** The transfection of anti-miR-130b or anti-miR-494 inhibited the migration and invasion of 5637 and T24 cells. **k** Predicted miR-130b/miR-494 binding sites in the 3′-UTR of *PTEN* mRNA by bioinformatics analysis. **l** Luciferase reporter assay was used to confirm the interaction between miR-130b/miR-494 and *PTEN* mRNA. **m** miR-130b and miR-494 decreased the protein expression level of PTEN, individually. Data are mean ± SD, n = 3

On the basis of the interaction of circSLC8A1 and miR-130b/miR-494, we next assessed the potential functional roles of miR-130b and miR-494 in bladder cancer. The result of qRT-PCR showed that miR-130b and miR-494 were up-regulated in bladder cancer tissues compared with normal bladder tissues (Fig. 4a and b, Table 2). Correlation analysis revealed a negative correlation between the expression of circSLC8A1 and miR-130b or miR-494 (Fig. 4c and d). Enforced expression of miR-130b and miR-494 significantly promoted 5737 and T24 proliferation (Fig. 4e and f). Transwell assays showed that over-expression of miR-130b or miR-494 significantly promoted the migration and invasion of 5637 and T24 cells (Fig. 4g and h). In contrast, transfection of miR-130b/miR-494 inhibitor obviously suppressed cell migration and invasion of 5637 and T24 cells (Figs. 4i and j). It has been reported that miRNAs post-transcriptionally regulate their target mRNA via sequence-guided recognition. According to TargetScan (http://www.targetscan.org/) and miRanda predictions, we found that miR-130b and miR-494 could both bind to the 3′-UTR region of *PTEN* (Fig. 4k). Next, luciferase reporter assays were performed to verify this interaction. The results showed that transfection of miR-130b or miR-494 mimics could strongly reduce the activity of a luciferase reporter carrying the wild-type *PTEN* 3′-UTR compared to mimic NC. Inversely, the mutated luciferase reporter was unaffected by over-expression of either miR-130b or miR-494 (Fig. 4l). Western blot assays demonstrated that miR-130b or miR-494 mimics could suppress the expression of PTEN (Fig. 4m). These results reveal that miR-130b/miR-494 can significantly promote the migration, invasion, and proliferation of bladder cancer through targeting *PTEN*.

**Table 2:** Correlations between the expression of miR-130b/miR-494 and clinicopathological features

	Cases	miR-130b expression		*p* Value	miR-494 expression		*p* Value
		Low	High		Low	High	
Age				1.000			0.952
< 55	11	3	8		2	9	
≥55	19	4	15		5	14	
Gender				1.000			0.914
Male	24	6	18		5	19	
Female	6	1	5		2	4	
T stage				**0.010**			< 0.001
pTa-T1	8	5	3		6	2	
pT2-T4	22	2	20		1	21	
Grade				**0.004**			0.047
Low	10	6	4		5	5	
High	20	1	19		2	18	
Lymph node metastasis				0.182			0.642
Absent	17	6	11		5	12	
Present	13	1	12		2	11	
Total	30	7	23		7	23	

*p* < 0.05 represents statistical significance

### circSLC8A1 regulates PTEN expression and inhibits bladder cancer progression via targeting miR-130b and miR-494

**Fig. 5:**
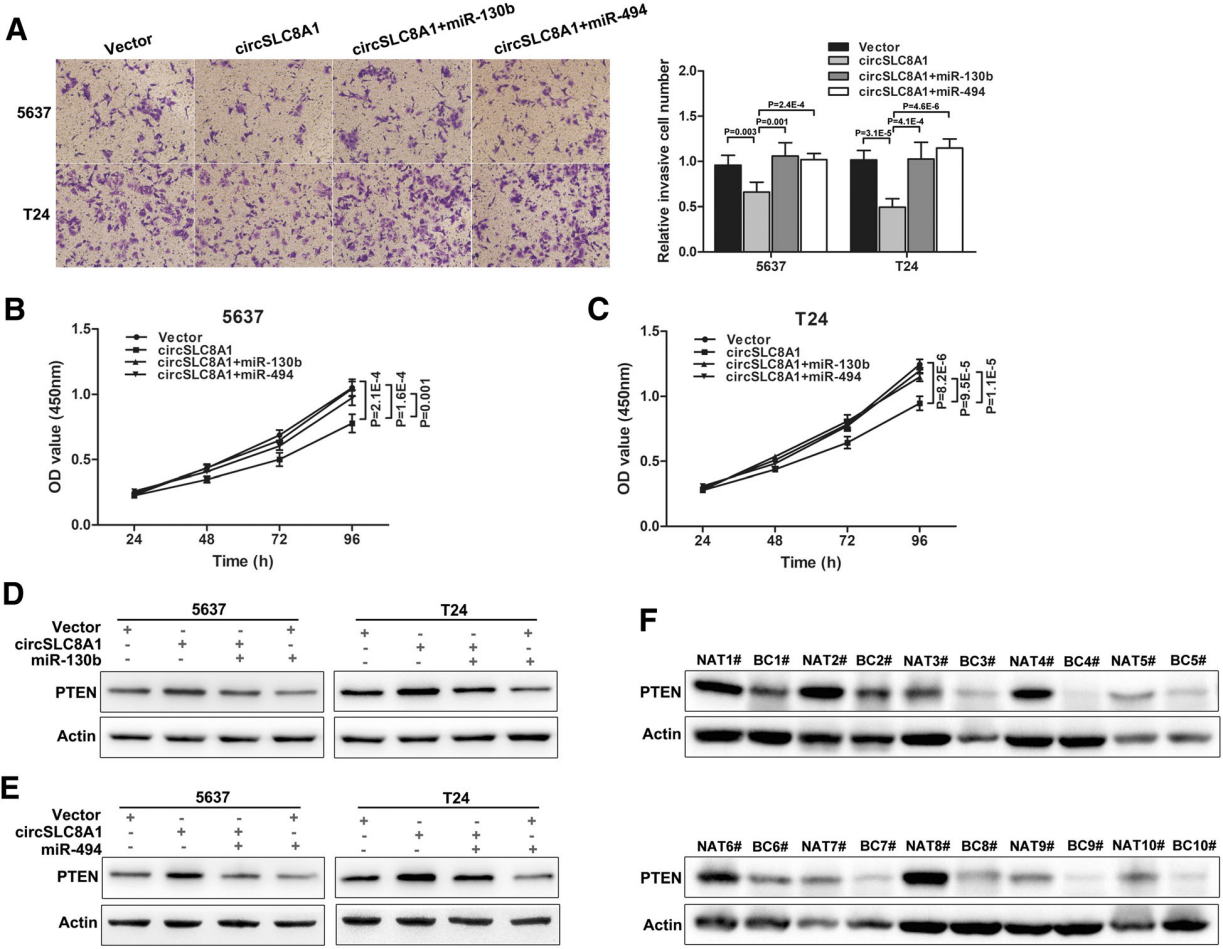
circSLC8A1 regulates PTEN expression and inhibits bladder cancer progression via targeting miR-130b and miR-494. **a**, **b** and **c** Transwell invasion and CCK-8 assays demonstrating that circSLC8A1 inhibited the invasion ability and proliferation of 5637 and T24 cells, and when co-transfected with miR-130b or miR-494, the inhibitory effect was reversed. **d** and **e** Western blot showed that miR-130b and miR-494 could partly decrease the protein expression level of PTEN which were promoted by circSLC8A1. **f** Western blot analysis of PTEN protein in 10 paired human bladder cancer specimens and normal adjacent tissue samples. Data are mean ± SD, n = 3

To assess whether circSLC8A1 inhibited the progression of bladder cancer cells via miR-130b and miR-494, rescue experiments were conducted by co-transfecting circSLC8A1 and miR-130b/miR-494 mimics into bladder cancer cells. Transwell matrigel invasion and CCK-8 assays in bladder cancer cells showed that overexpression of circSLC8A1 led to inhibition of the invasion and proliferation capabilities, but this effect could be partly attenuated by ectopic expression of miR-130b or miR-494 (Fig. 5a, b and c). Moreover, we found that the expression of PTEN was significantly decreased in the bladder cancer cells co-transfected with circSLC8A1 plasmids and miR-130b/miR-494 mimics, compared with the cells transfected with circSLC8A1 alone (Fig. 5d and e), which agreed with the results of cell function. Additionally, the expression levels of PTEN protein were strikingly lower in bladder cancer specimens compared to adjacent noncancerous specimens (Fig. 5f). The above results demonstrated that circSLC8A1 suppressed bladder cancer progression via sponging miR-130b/miR-494 and regulated PTEN expression.

### circSLC8A1 suppresses the growth of bladder cancer tumor in vivo

**Fig. 6:**
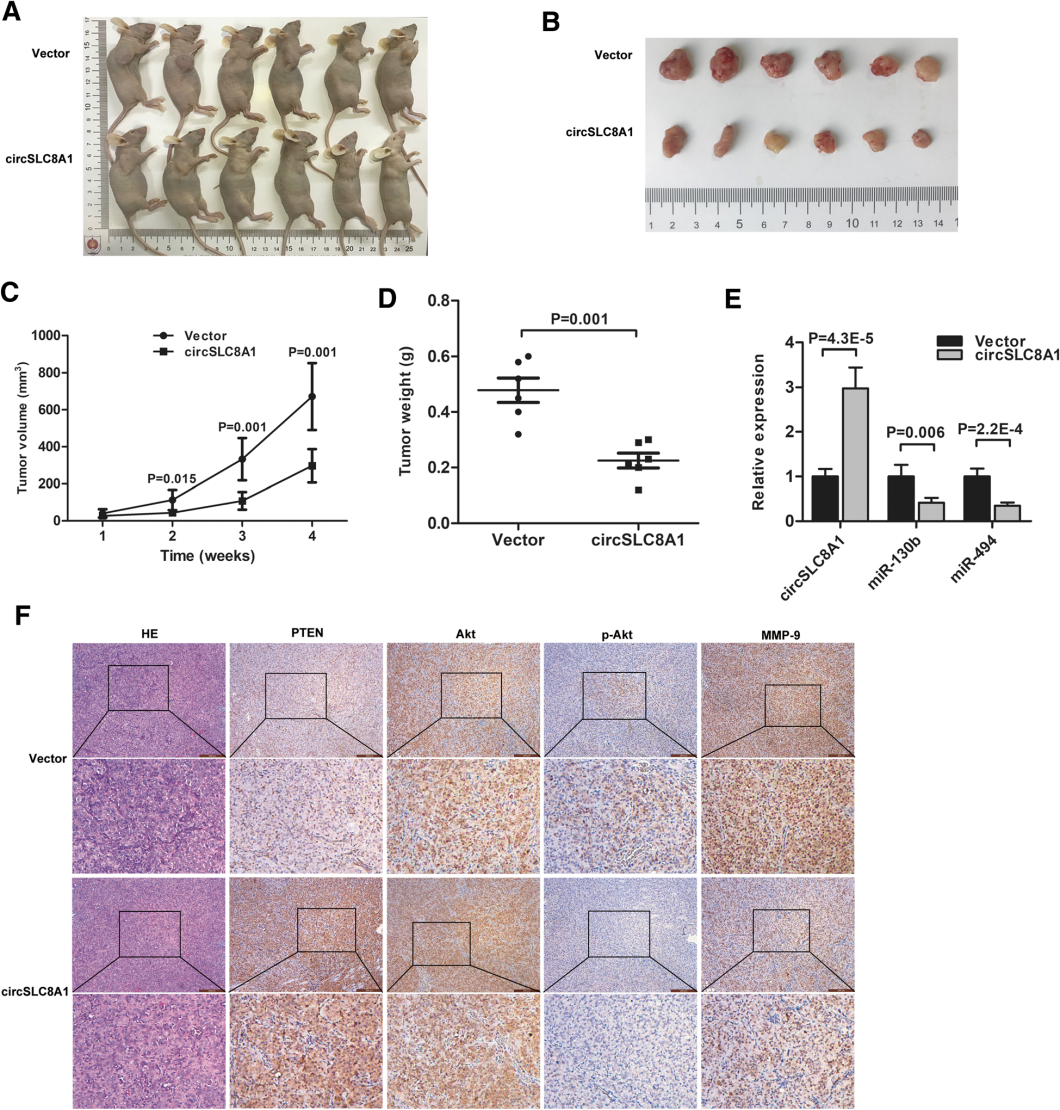
circSLC8A1 suppresses the growth of bladder cancer tumor in vivo. **a** Hypodermic injection of T24 cells stably transfected with circSLC8A1 or control vector into nude mice established subcutaneous xenograft tumors (*n* = 6 for each group). **b** Representation picture of tumor formation of the xenograft in nude mice. **c** and **d** Compared with the vector group, the tumor growth rate and weight significantly decreased in circSLC8A1-treated nude mice. **e** circSLC8A1, miR-130b, and miR-494 levels were detected by qRT-PCR in tumor tissues. **f** The expressions of PTEN, AKT, p-AKT, and MMP9 were measured using IHC staining in the xenografts

To investigate whether over-expression of circSLC8A1 regulates tumor growth in vivo, T24 cells stably transfected with circSLC8A1 or control vector were injected subcutaneously into BALB/c nude mice. The tumor volumes were measured weekly after injection. Compared with the control group, the circSLC8A1 over-expression group significantly reduced the growth rate and tumor weight (Fig. 6c and d). In the circSLC8A1 over-expression group, the level of circSLC8A1 was upregulated, whereas the levels of miR-130b and miR-494 were downregulated according to the qRT-PCR results (Fig. 6e). IHC analysis showed that the expression of PTEN was elevated in the tumors by over-expressing circSLC8A1 (Fig. 6f). It is known that PTEN is the negative regulator of the PI3K/Akt pathway, and MMP-9 protein which is a key factor of cancer invasion is regulated by the PI3K/Akt signaling pathway [24, 25]. IHC analysis revealed that the expression of phosphorylated Akt (p-Akt) and MMP-9 was inhibited by over-expressing circSLC8A1 (Fig. 6f). These results demonstrate that over-expression of circSLC8A1 efficiently inhibits the growth of bladder cancer in vivo.

## Discussion

In this study, we first confirmed that circSLC8A1 was an important circRNA frequently down-regulated in bladder cancer tissue, and a lower expression of circSLC8A1 was positively correlated with the histological grade and tumor stage. Second, we demonstrated that circSLC8A1 acted as a sponge of miR-130b/miR-494 in suppressing bladder cancer progression via regulating PTEN. Given the importance of the PTEN signaling in bladder cancer progression, our results revealed the function, mechanism and clinical implication of circSLC8A1 in human bladder cancer.

Increasing evidence suggests that circRNAs are not simply the junk-products in pre-mRNA splicing [26]. With the advent of next-generation sequencing, numerous of circRNAs have been identified from various animal genomes, and many of them are highly stable and abundantly expressed. During the past years, a group of circRNAs has been reported to be dysregulated in diverse cancer types, such as hepatocellular carcinoma [22], esophageal squamous cell carcinoma [27], oral cancer [28], bladder cancer [29], and other types of cancer [30]. Many studies have confirmed that stable transcripts with a host of miRNA-binding sites or miRNA response elements (MREs) could function as miRNA sponges [6], and circRNAs are found to be enriched in functional miRNA binding sites [17].

CircRNAs, which have been regarded as miRNA sponges, have some common characteristics. They are derived from one or more exons of known protein-coding genes through back-splicing [6]. Moreover, the subcellular location of these circRNAs is predominantly in the cytoplasm, which occupies the same space as miRNA. Besides, circRNAs harboring more predictive putative miRNA binding sites could be potential candidates for miRNA sponges [22]. It should be noted that not all the circRNAs can act as miRNA sponges. CircRNAs with small length, which are apparently not suitable for miRNA sponges, can be packaged into exosomes and function as cancer biomarkers [31]. Intronic and exon–intron RNAs, which mainly localize in nucleus, have been reported to regulate their parental genes expression via specific RNA–RNA interaction [32].

Due to the sequence conservation, biological stability and tissue specificity of circRNAs, they are thought to be promising biomarkers, therapy targets, and may exert potential functions in the regulation of gene expression [33, 34]. To better understand the regulatory mechanism of circRNA in this study, we focused on circSLC8A1 which was down-regulated in bladder cancer first. We identified that circSLC8A1, which consists of one exon (1832 bp) from the *SLC8A1* gene, mainly located in the cytoplasm. Our miRNA-targeting prediction analysis showed that circSLC8A1 contained multiple miRNA binding sites, suggesting that circSLC8A1 may act as a miRNA sponge. Our results revealed that circSLC8A1 interacted with both miR-130b and miR-494 in bladder cancer cells.

Next, we found that the circSLC8A1-mediated inhibition of invasion and proliferation could be attenuated by enforced overexpression of miR-130b or miR-494. Furthermore, bioinformatics prediction and luciferase reporter assay revealed that miR-130b and miR-494 were critical negative regulators of the PTEN pathway. Lastly, circSLC8A1 could regulate PTEN expression by sponging miR-130b and miR-494. Upregulated expression of circSLC8A1 led to the activation of PTEN signaling in bladder cancer, which could be alleviated by miR-130b and miR-494.

*PTEN* has been widely known as a tumor suppressor gene, and *PTEN* mutation or deletion is frequently noted in many cancers [35]. The most known function of PTEN is as a negative regulator of the PI3K/Akt pathway, which is a crucial signal transduction pathway for cancer cell growth [36]. Moreover, an integrated study of 131 high grade muscle-invasive bladder cancer samples has revealed dysregulation of the PI3K/Akt signaling pathway in 72% of cases [37]. The forced expression of PTEN reduced Akt phosphorylation as well as suppressed cell proliferation, migration and invasion [38, 39]. Additionally, MMP-9 protein, which is a key factor of bladder cancer invasion, is regulated by the PI3K/Akt signaling pathway [25]. IHC analysis of xenografts in our study revealed that the expressions of p-Akt and MMP-9 were inhibited by over-expressing circSLC8A1.

## Conclusions

In conclusion, the results of our study demonstrate that circSLC8A1 is down-regulated in bladder cancer tissues and cell lines, and it is capable of functioning as a sponge for miR-130b and miR-494 to regulate the expression of PTEN. Moreover, we also demonstrate that over-expression of circSLC8A1 can effectively inhibit the progression of bladder cancer via targeting the miR-130b, miR-494/PTEN axis. Our results not only explain the mechanisms of circRNA in regulating bladder cancer cell progression, but also provide a potential biomarker and therapeutic target for the management of bladder cancer.

## Additional files

Additional file 1: **Figure S1.** Five candidate circRNAs were validated using qRT-PCR in bladder cancer tissues and matched adjacent normal tissues. **A** to **E** The expression levels of the five circRNAs were validated by qRT-PCR in 20 patients. **F** The comparison between RNA-sequencing data and qRT-PCR results. The vertical axis showed the mean of the fold change (log2 transformed) of each circRNA measured by qRT-PCR and RNA-sequencing, respectively. (JPG 1781 kb)

Additional file 2: **Figure S2.** Cell migration and invasion abilities of 5637 and T24 cells transfected with si circSLC8A1-2 or siNC were evaluated. **A** The effect of si circSLC8A1–2 on cell migration capability was evaluated by wound healing assay in 5637 and T24 cells, respectively. **B** and **C** Cell migration and invasion abilities of 5637 and T24 cells transfected with si circSLC8A1–2 or siNC were evaluated by transwell migration and invasion assays. (JPG 5484 kb)

Additional file 3: **Table S1.** Detailed information of five candidate circRNAs. (DOCX 14 kb)

Additional file 4: **Table S2.** Primers and RNA sequences used in this study. (DOCX 19 kb)

## Data Availability

Please contact the corresponding author for all data requests.

## Abbreviations

3′-UTR: 3′ untranslated region
CCK-8: Cell Counting Kit-8
circRNA: circular RNA
FBS: Fetal bovine serum
H&E: Hematoxylin and eosin
IHC: Immunohistochemistry
miRNA: microRNA
qRT-PCR: Quantitative reverse transcription polymerase chain reaction
siRNA: small interfering RNA
β-gal: β-galactosidase
