# Supplementary figures and images for: Circular RNA circSLC8A1 acts as a sponge of miR-130b/miR-494 in suppressing bladder cancer progression via regulating PTEN
Source: Mol Cancer. 2019 Jun 22;18:111. doi: 10.1186/s12943-019-1040-0 (PMC6588875; doi:10.1186/s12943-019-1040-0)

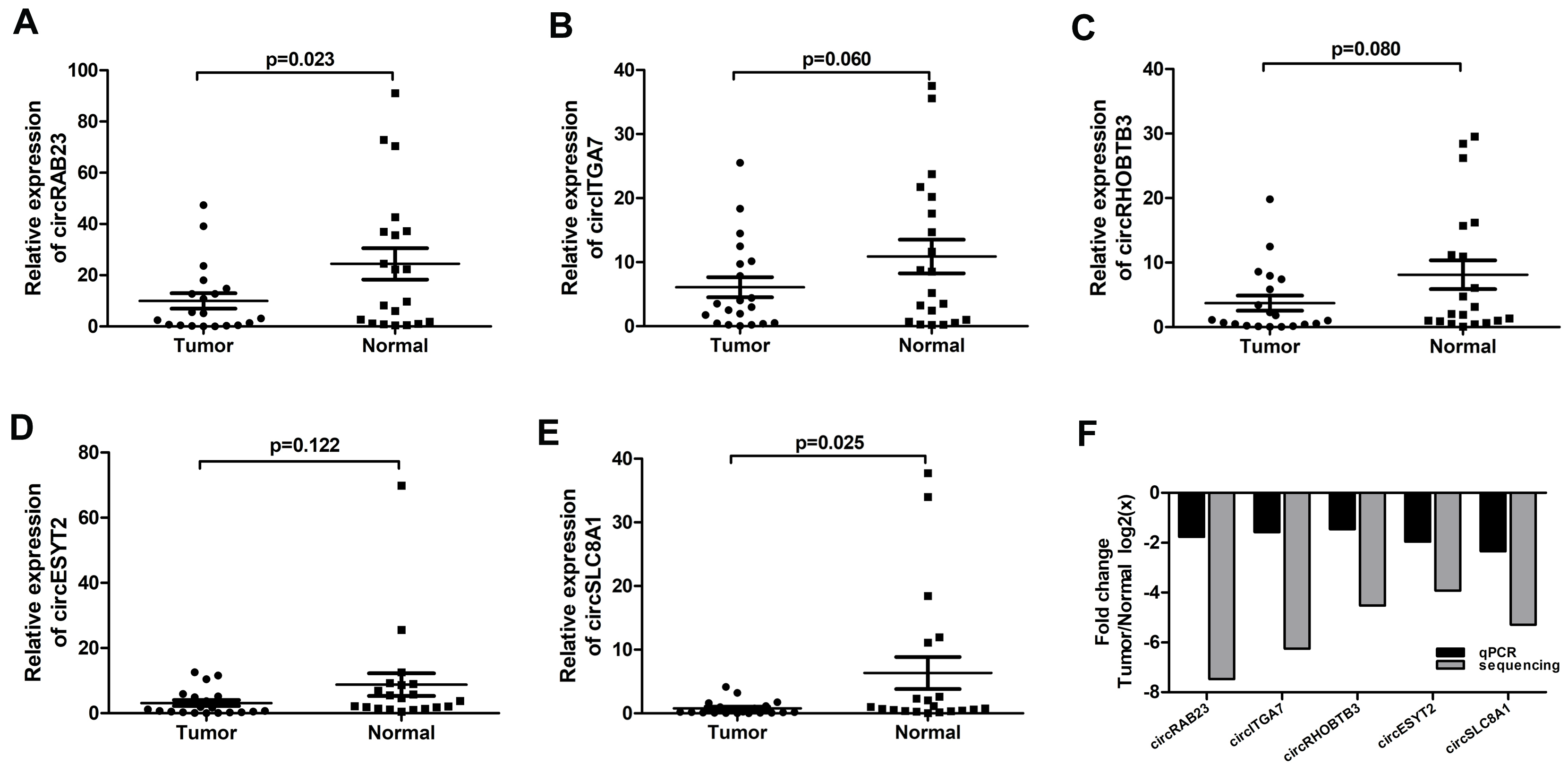

Supplement: Supplementary file 1 — Figure S1. Five candidate circRNAs were validated using qRT-PCR in bladder cancer tissues and matched adjacent normal tissues. A to E The expression levels of the five circRNAs were validated by qRT-PCR in 20 patients. F The comparison between RNA-sequencing data and qRT-PCR results. The vertical axis showed the mean of the fold change (log2 transformed) of each circRNA measured by qRT-PCR and RNA-sequencing, respectively. (JPG 1781 kb) [file 12943_2019_1040_MOESM1_ESM.jpg]

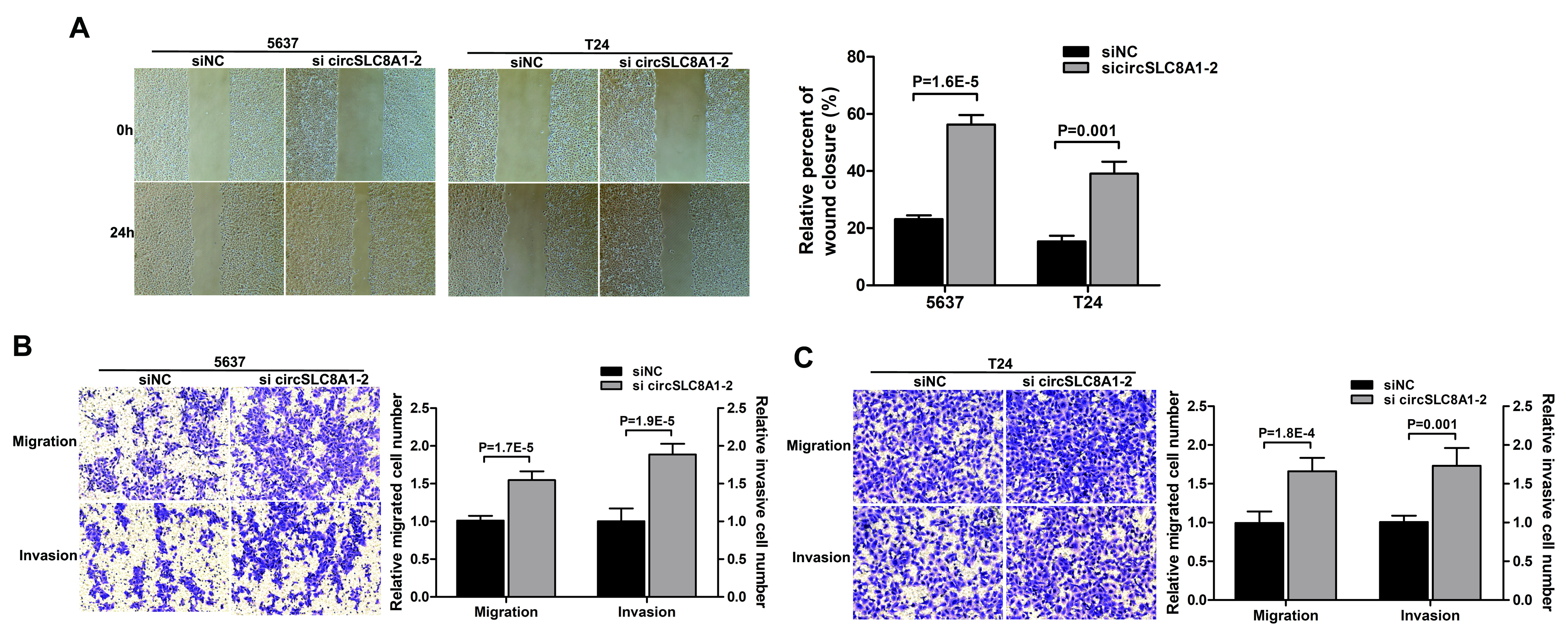

Supplement: Supplementary file 2 — Figure S2. Cell migration and invasion abilities of 5637 and T24 cells transfected with si circSLC8A1-2 or siNC were evaluated. A The effect of si circSLC8A1–2 on cell migration capability was evaluated by wound healing assay in 5637 and T24 cells, respectively. B and C Cell migration and invasion abilities of 5637 and T24 cells transfected with si circSLC8A1–2 or siNC were evaluated by transwell migration and invasion assays. (JPG 5484 kb) [file 12943_2019_1040_MOESM2_ESM.jpg]
