# Supplementary material for: Circular RNA circSLC8A1 acts as a sponge of miR-130b/miR-494 in suppressing bladder cancer progression via regulating PTEN
Source: Mol Cancer. 2019 Jun 22;18:111. doi: 10.1186/s12943-019-1040-0 (PMC6588875; doi:10.1186/s12943-019-1040-0)
Supplement: Supplementary file 3 — Table S1. Detailed information of five candidate circRNAs. (DOCX 14 kb) [file 12943_2019_1040_MOESM3_ESM.docx]

**Supplementary Table 1** Detailed information of five candidate circRNAs

| Name | Circ_loci | Strand | Genomic length | Spliced length |
| --- | --- | --- | --- | --- |
| circRAB23 | chr6:57058639-57075243 | - | 16604 | 639 |
| circITGA7 | chr12:56094682-56094938 | - | 256 | 256 |
| circRHOBTB3 | chr5:95091099-95099324 | + | 8225 | 479 |
| circESYT2 | chr7:158552176-158557544 | - | 5368 | 495 |
| circSLC8A1 | chr2:40655612-40657444 | - | 1832 | 1832 |
