# Supplementary material for: Circular RNA circSLC8A1 acts as a sponge of miR-130b/miR-494 in suppressing bladder cancer progression via regulating PTEN
Source: Mol Cancer. 2019 Jun 22;18:111. doi: 10.1186/s12943-019-1040-0 (PMC6588875; doi:10.1186/s12943-019-1040-0)
Supplement: Supplementary file 4 — Table S2. Primers and RNA sequences used in this study. (DOCX 19 kb) [file 12943_2019_1040_MOESM4_ESM.docx]

**Supplementary Table 2** Primers and RNA sequences used in this study

| **List of oligonucleotide sequences** | **5'--> 3'** |
| --- | --- |
| **Primers for PCR** |  |
| circRAB23 Forward | CTGAGGCACTGGCAAAAAGG |
| circRAB23 Reverse | TTGGAGCTGAAATGGTTTCTGT |
| circITGA7 Forward | ACAATTTGGGTTCTGCCAGC |
| circITGA7 Reverse | TGCCTTGCCTCATATCGGTG |
| circRHOBTB3 Forward | AGGCAACCCACCATTACGAG |
| circRHOBTB3 Reverse | TGTGACGCTTCAGCCTTTAAGA |
| circESYT2 Forward | TGCCAAATGCGTCAAACCTC |
| circESYT2 Reverse | AGGGGTCTGACTTTCCCTTG |
| circSLC8A1 Forward | ATCGAAGGGACTGCCAGAGG |
| circSLC8A1 Reverse | GGTGAAAGACTTAATCGCCGC |
| linear-SLC8A1 Forward | AAACCATCGAAGGGACTGC |
| linear-SLC8A1 Reverse | TTACCTTGACTGATATTGTTTTGACA |
| GAPDH Forward | CAATGACCCCTTCATTGACC |
| GAPDH Reverse | TTGATTTTGGAGGGATCTCG |
| divergent-GAPDH Forward | GAAGGTGAAGGTCGAGTC |
| divergent-GAPDH Reverse | GAAGATGGTGATGGGATTTC |
| hsa-miR-129 RT | CTCAACTGGTGTCGTGGAGTCGGCAATTCAGTTGAGGCAAGCCC |
| hsa-miR-129 Forward | ACACTCCAGCTGGGCTTTTTGCGGTCTGG |
| hsa-miR-130b RT | CTCAACTGGTGTCGTGGAGTCGGCAATTCAGTTGAGATGCCCTT |
| hsa-miR-130b Forward | ACACTCCAGCTGGGCAGTGCAATGATGAAA |
| hsa-miR-133b RT | CTCAACTGGTGTCGTGGAGTCGGCAATTCAGTTGAGTAGCTGGT |
| hsa-miR-133b Forward | ACACTCCAGCTGGGTTTGGTCCCCTTCAAC |
| hsa-miR-136 RT | CTCAACTGGTGTCGTGGAGTCGGCAATTCAGTTGAGTCCATCAT |
| hsa-miR-136 Forward | ACACTCCAGCTGGGACTCCATTTGTTTTGAT |
| hsa-miR-140b RT | CTCAACTGGTGTCGTGGAGTCGGCAATTCAGTTGAGCCGTGGTT |
| hsa-miR-140b Forward | ACACTCCAGCTGGGTACCACAGGGTAGAA |
| hsa-miR-197 RT | CTCAACTGGTGTCGTGGAGTCGGCAATTCAGTTGAGGCTGGGTG |
| hsa-miR-197 Forward | ACACTCCAGCTGGGTTCACCACCTTCTCCA |
| hsa-miR-494 RT | CTCAACTGGTGTCGTGGAGTCGGCAATTCAGTTGAGGAGGTTTC |
| hsa-miR-494 Forward | ACACTCCAGCTGGGTGAAACATACACGGGA |
| unified reverse primer | TGGTGTCGTGGAGTCG |
| U6 Forward | CTCGCTTCGGCAGCACA |
| U6 Reverse | AACGCTTCACGAATTTGCGT |
| **siRNAs** |  |
| si NC sense | UUCUCCGAACGUGUCACGUTT |
| si NC antisense | ACGUGACACGUUCGGAGAATT |
| si circSLC8A1-1 sense | AUUGUUAGGUUGUGACAGUTT |
| si circSLC8A1-1 antisense | ACUGUCACAACCUAACAAUTT |
| si circSLC8A1-2 sense | GAUGAAAUUGUUAGGUUGUTT |
| si circSLC8A1-2 antisense | ACAACCUAACAAUUUCAUCTT |
| **FISH probes** |  |
| circSLC8A1 | AACTGTCACAACCTAACAATTTCAT |
| miR-130b | ATGCCCTTTCATCATTGCACTG |
| miR-494 | GAGGTTTCCCGTGTATGTTTCA |
| **Biotinylated probes** |  |
| biotin-NC | GTGTAACACGTCTATACGCCCA |
| biotin-circSLC8A1-1 | ACTGTCACAACCTAACAATTTCATCATTCT |
| biotin-circSLC8A1-2 | CCAACTGTCACAACCTAACAATTTCATCAT |
| biotin-NC mimics sense | UUCUCCGAACGUGUCACGUTT |
| biotin-NC mimics antisense | ACGUGACACGUUCGGAGAATT |
| biotin-miR-130b mimics sense | CAGUGCAAUGAUGAAAGGGCAU |
| biotin-miR-130b mimics antisense | GCCCUUUCAUCAUUGCACUGUU |
| biotin-miR-494 mimics sense | UGAAACAUACACGGGAAACCUC |
| biotin-miR-494 mimics antisense | GGUUUCCCGUGUAUGUUUCAUU |
| **miRNA mimics and inhibitors** |  |
| mimics NC sense | UUCUCCGAACGUGUCACGUTT |
| mimics NC antisense | ACGUGACACGUUCGGAGAATT |
| miR-130b mimics sense | CAGUGCAAUGAUGAAAGGGCAU |
| miR-130b mimics antisense | GCCCUUUCAUCAUUGCACUGUU |
| miR-494 mimics sense | UGAAACAUACACGGGAAACCUC |
| miR-494 mimics antisense | GGUUUCCCGUGUAUGUUUCAUU |
| anti miR-NC | UCUACUCUUUCUAGGAGGUUGUGA |
| anti miR-130b | AUGCCCUUUCAUCAUUGCACUG |
| anti miR-494 | GAGGUUUCCCGUGUAUGUUUCA |
